# The Effect of Monetary Incentives on Health Care Social Media Content: Study Based on Topic Modeling and Sentiment Analysis

**DOI:** 10.2196/44307

**Published:** 2023-05-11

**Authors:** Negar Maleki, Balaji Padmanabhan, Kaushik Dutta

**Affiliations:** 1 School of Information Systems and Management University of South Florida Tampa, FL United States

**Keywords:** health care analytics, social media, incentive mechanisms, content analysis, contrastive topic modeling

## Abstract

**Background:**

While there is high-quality online health information, a lot of recent work has unfortunately highlighted significant issues with the health content on social media platforms (eg, fake news and misinformation), the consequences of which are severe in health care. One solution is to investigate methods that encourage users to post high-quality content.

**Objective:**

Incentives have been shown to work in many domains, but until recently, there was no method to provide financial incentives easily on social media for users to generate high-quality content. This study investigates the following question: What effect does the provision of incentives have on the creation of social media health care content?

**Methods:**

We analyzed 8328 health-related posts from an incentive-based platform (Steemit) and 1682 health-related posts from a traditional platform (Reddit). Using topic modeling and sentiment analysis–based methods in machine learning, we analyzed these posts across the following 3 dimensions: (1) emotion and language style using the IBM Watson Tone Analyzer service, (2) topic similarity and difference from contrastive topic modeling, and (3) the extent to which posts resemble clickbait. We also conducted a survey using 276 Amazon Mechanical Turk (MTurk) users and asked them to score the quality of Steemit and Reddit posts.

**Results:**

Using the Watson Tone Analyzer in a sample of 2000 posts from Steemit and Reddit, we found that more than double the number of Steemit posts had a confident language style compared with Reddit posts (77 vs 30). Moreover, 50% more Steemit posts had analytical content and 33% less Steemit posts had a tentative language style compared with Reddit posts (619 vs 430 and 416 vs 627, respectively). Furthermore, more than double the number of Steemit posts were considered joyful compared with Reddit posts (435 vs 200), whereas negative posts (eg, sadness, fear, and anger) were 33% less on Steemit than on Reddit (384 vs 569). Contrastive topic discovery showed that only 20% (2/10) of topics were common, and Steemit had more unique topics than Reddit (5 vs 3). Qualitatively, Steemit topics were more informational, while Reddit topics involved discussions, which may explain some of the quantitative differences. Manual labeling marked more Steemit headlines as clickbait than Reddit headlines (66 vs 26), and machine learning model labeling consistently identified a higher percentage of Steemit headlines as clickbait than Reddit headlines. In the survey, MTurk users said that at least 57% of Steemit posts had better quality than Reddit posts, and they were at least 52% more likely to like and comment on Steemit posts than Reddit posts.

**Conclusions:**

It is becoming increasingly important to ensure high-quality health content on social media; therefore, incentive-based social media could be important in the design of next-generation social platforms for health information.

## Introduction

### Background

Seeking online health information, also called “interactive health communication” [1], has become quite common. Factors, including age, gender, income [2], and cultural barriers [3], as well as online facilitators, including real-time interaction, privacy features, and archived health information [2], lead individuals to use online health information in their daily lives to often seek information about their own health conditions. When used appropriately, online health information can help make better decisions for the benefit of patients, families, consumers, and caregivers [4]. Moreover, health information accessibility on the internet encourages individuals to contribute health information from professional medical websites.

As of July 2022, the global social media user base reached 59% of the world’s total population [5]. Social media is omnipresent, evolving quickly, and increasingly affecting people’s lives and health behaviors. The idea of Medicine 2.0 was developed in response to the advent of Web 2.0 to accommodate a better internet environment for social networking, collaboration, participation, apomediation, and openness [6]. Social media are key platforms for the concepts of Web 2.0 and Medicine 2.0 within these topics and have the potential to be extremely effective tools for enticing and empowering users who are looking for health information [6-8].

While social media could provide high-quality health information [9], low-quality pieces can also be found on these platforms (eg, online misinformation) [10]. Distinguishing high-quality health information from low-quality health information is a major problem on social media platforms and remains an issue that has not been sufficiently addressed in health care communities. Recently, incentive-driven social media platforms are evolving, in which users’ activities (ie, posting content) are compensated based on how users on the platform react to such content. One possible solution could therefore involve the use of such Web 3.0 platforms that are based on monetary incentives. This study explores this possibility by comparing health care posts across 2 social media platforms that differ in terms of incentives. Steemit [11] is a platform that rewards users for participation on the site, whereas Reddit is a platform that does not offer incentives to users for publishing content.

One of the key questions behind this broader effort is how the implemented incentive mechanism affects the kind of content generated on these platforms. While an increasing body of work in the literature [12-19] is examining incentive-based social media from different perspectives, there is very little work to date that has compared incentive-based and nonincentive-based platforms on the quality and characteristics of posts. To the best of our knowledge, this is the first study to cover this gap systematically.

While there are no direct comparisons to the work performed in this study, there is growing interest in examining broader issues related to content quality in social media. Social media users have various backgrounds, motivations, opinions, and experience levels. As a result, the quality of user-generated content (eg, posts) on social media varies greatly [20]. Evaluating content quality on social media aids in the identification and promotion of high-quality information over low-quality information. This evaluation, therefore, can help with the problem of misinformation. However, there are challenges in determining content quality for social media, while information regarded as high quality by one user may be judged as low quality by another user [21].

A recent study suggested the use of content labeling in social media to deal with issues, such as misinformation and misleading content, which may impact anything from voting to personal health; however, those who seek to spread misinformation always try to find new tactics, methods, and formats to pursue their goals [22]. This study investigates the incentive mechanism as another factor that can deal with these issues. Our findings reveal that health-related posts on a social media platform with an incentive mechanism are different in a systematic way from health-related posts on a traditional social media platform. While our work here is primarily exploratory in nature, the results point to the potential of investigating the use of incentives to help improve health-related content on social media.

In this study, we identified the following 3 dimensions specific to social media that can be used for such a comparison: (1) contrastive topics, (2) emotion and language style, and (3) whether the content is “clickbait.” Among these, the idea of contrastive topics [23] is relatively new. We present essential differences in these dimensions that may have significant implications. We found that the incentive mechanism in play likely motivates posts that are more informational than personal. We also found differences in emotion, tone, and the extent to which posts are created with potential *clickbait* content. While this is still early in the evolution of newer incentive-based social media, the results suggest that there is an opportunity to study user behavior on these platforms and to use some of those findings to re-engineer current platforms toward directions that can help alleviate concerns, such as misinformation, echo chambers, and other social ills, which we are observing for the more common platforms widely in use at present.

### Objective

The main objective of this study is to understand if there is any difference in health-related content across social media platforms with and without monetary incentives. For the traditional (no incentive) platform, we used Reddit, and for the incentive-driven platform, we used the blockchain social media platform Steemit. Though the basic structure of Reddit and Steemit is similar (Steemit was originally developed by modeling Reddit), we expect to see some differences in content on these 2 platforms in part due to the incentive mechanism in place. Past research [24,25] has performed such comparisons using topic modeling. We followed the same approach of content comparison by topic modeling. However, we used the approach presented previously [23] that focused on *contrastive* topic modeling, which has been specifically designed to bring out similarities and differences between corpora. Additionally, we compared posts across Steemit and Reddit using the emotion and language style expressed in the content. For this, we used the Watson Tone Analyzer [26]. Moreover, we examined both groups based on the likelihood of content being “clickbait” (using both a machine learning approach [27] and a manual approach). The hypothesis here was that having an incentive mechanism might encourage users to use clickbait-related ideas in their titles or posts to gain more user engagement. Thus, it can also be viewed as a surrogate (and noisy) measurement for user engagement. Finally, the quality of posts within the Steemit and Reddit platforms was examined through an online survey, with the hypothesis that an incentive mechanism makes authors post high-quality content. We describe the procedures and results of comparisons across each of these dimensions (topic modeling, emotion and language style, clickbait, and content quality).

## Methods

### Data Collection

We introduce the data sets that we used in our work. SteemOps is a data set [28], which contains 10 key types of Steemit operations organized into the following 3 subdatasets: (1) the social-network operation data set, (2) the witness-election operation data set, and (3) the value-transfer operation data set. The data were collected from March 24, 2016, 4:05 PM to December 01, 2019, 12 AM.

The main subdataset we used in this paper is the social-network operation data set, consisting of 3 operational keys: comment, vote, and custom-json. The comment operation consists of 5 fields (Table 1). According to a previous report [28], a new post is indicated when both parent-author and parent-permlink fields are empty. When these 2 fields are not empty, it represents a comment to a post/comment. Each post in Steemit remains active for 7 days, so each time the author makes any changes to the post, the post is recorded as a new post in the data set. Moreover, each post’s permlink is unique, so considering all of these factors, the data set consists of 17,805,355 new posts.

**Figure 1:**
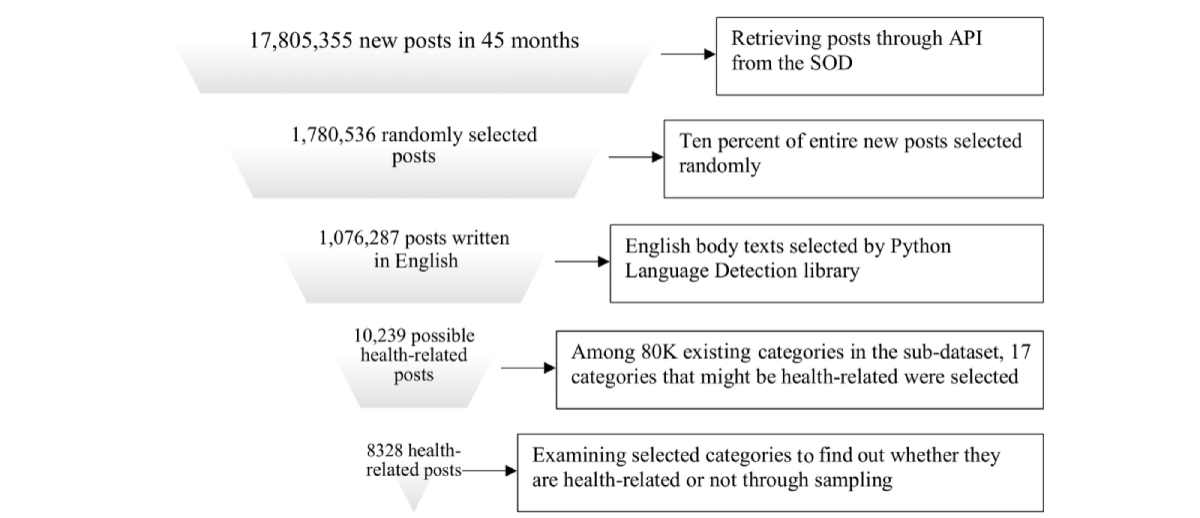
Final data set process on the Steemit platform. API: application programming interface; SOD: social-network operation data set.

The Steemit platform offers an interactive application programming interface (API) for researchers to parse the data. However, just retrieving the full information considering some API restrictions would have taken approximately 38 days in total, so we retrieved a random 10% sample of this data set (approximately 1.7 million new posts) for further analysis. Among the 10% data, we used posts written in English (1,076,287 posts remained) for easier and more consistent comparison and analysis. Figure 1 shows the process of obtaining the final data set for Steemit analysis.

Reddit also provides an API for accessing any possible information. Moreover, the Reddit API leverages finding health-related content by giving access to health subreddits. We retrieved health-related content in specific health subreddits with the restriction of getting a certain amount of data in each loop. After several attempts of retrieving posts for each subreddit, we ended up having 10,096 Reddit posts in total. However, we had a lot of reiterative content because of the API restriction. After removing reiterative posts and filtering English content, we had 1682 health-related posts from the Reddit platform for analysis. The following section explains obtaining health-related posts on Reddit and retrieving health-related posts on Steemit.

**Table 1:** Schema of the comment operation [28].

Field name	Description
block_no	The block recording this operation.
parent_author	The author that the comment is being submitted to.
parent_permlink	The specific post that the comment is being submitted to.
author	The author of the post/comment being submitted (account name).
permlink	The unique string identifier for the post, which is linked to the author of the post.

### Finding Health-Related Posts

Finding health-related keywords that could cover health-related words in social media posts is challenging. Many social media users who write posts in the health category are likely not physicians, and they may, therefore, use incorrect terminology (making formal keywords alone insufficient). On the other hand, some people may use health-related words while not planning to post in the health category. To address this issue, we decided to use the “parent permlink” or “category” of posts, which would be the first tag each author chooses for the post. However, if the first tag is among the Steemit popular tags (a list of popular tags has been provided by Steemit), it remains the same; otherwise, the Steemit platform puts different words as the “parent permlink” or “category” [29].

Although choosing the appropriate tags is essential to authors as they are rewarded if they do it correctly, many posts are categorized in inappropriate categories. To solve this problem and see which categories are more relevant to health, we counted how many times each “category” repeated in all the English posts, and selected any of them that may be relevant to the health category and that had more than 100 posts within. The second column in Table 2 shows the number of English posts of any possible relevant “category.” Then, we obtained a sample set randomly, which included 700 samples from all the posts written in English, and we read them all individually to check whether they were relevant to “health.” The sampling results are also presented in Table 2 (columns 3-6).

As we can see in Table 2, some categories have many irrelevant posts within, so by removing these categories, we can see a great improvement in the accuracy. These irrelevant categories are “Fitness,” “Fruit,” “Lifestyle,” “Beauty,” “Tips,” “Energy,” and “Vegan.” Table 3 provides a summary of the sample set before and after removing the irrelevant categories.

**Figure 2:**
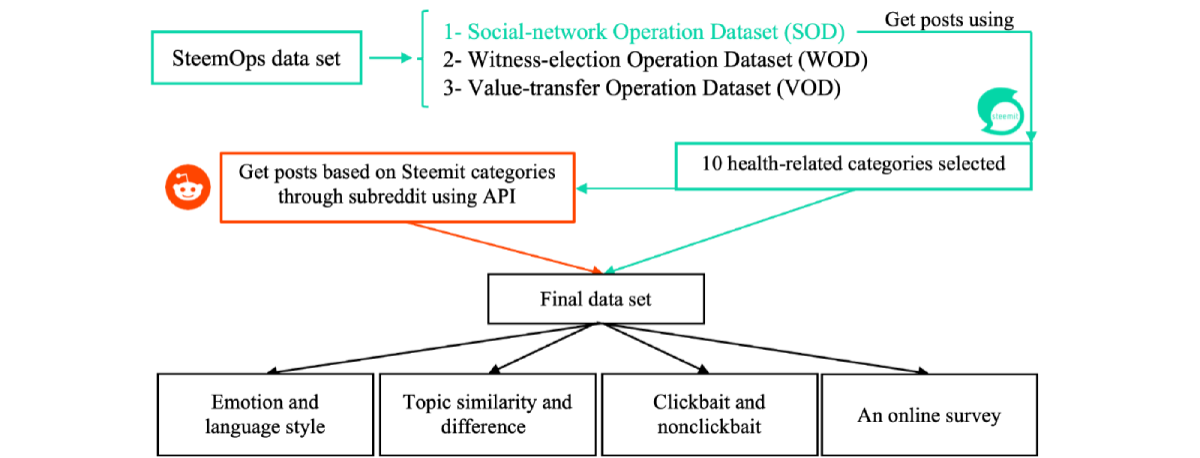
Methodology. API: application programming interface.

Unlike Steemit, Reddit does not have an incentive system that encourages writers to include a category when they post. However, there is another criterion that functions similar to the Steemit category. A subreddit [30] is a distinct online community dedicated to a certain topic about which people post. As a result, we chose health-related posts on Reddit based on the categories we discovered on Steemit. Therefore, we included all subreddits under the terms “health,” “health care,” “yoga,” “medicine,” “meditation,” “cancer,” “healthy,” “drugs,” “diet,” and “medical” besides those similar to these subreddits. The Reddit API enables the retrieval of particular posts in the target subreddit; thus, this method was used to retrieve health-related Reddit posts. Figure 2 provides a brief summary of how we gathered the data set and for which terms we plan to compare Steemit and Reddit.

**Table 2:** Steemit potential categories with the number of English posts (column 2) and the findings for the Steemit sample set (columns 3-6).

Category	Number of English posts (N=10,239)	Number of sample posts in the category (N=700)	Number of irrelevant posts (N=243)	Number of relevant posts (N=457)	Match percentage
Health^a^	7078	407	73	334	82.06
Fitness	592	64	40	24	37.50
Fruit	129	12	7	5	41.67
Health care^a^	145	7	0	7	100.00
Yoga^a^	175	19	6	13	68.42
Medicine^a^	133	7	2	5	71.43
Meditation^a^	135	12	1	11	91.67
Cancer^a^	114	6	0	6	100.00
Healthy^a^	169	11	0	11	100.00
Lifestyle	410	49	45	4	8.16
Beauty	264	33	26	7	21.21
Tips	110	9	8	1	11.11
Drugs^a^	122	14	7	7	50.00
Diet^a^	115	10	0	10	100.00
Medical^a^	142	8	3	5	62.50
Energy	114	7	6	1	14.29
Vegan	292	25	19	6	24.00

^a^Relevant category based on the match percentage. Overall, the relevant categories had 8328 English posts, 501 sample posts in the category, 92 irrelevant posts, 409 relevant posts, and a match percentage of 81.64%.

**Table 3:** Steemit sample set summary.

Variable	Average number of relevant posts	Average number of posts	Average match percentage	Total population, n	Match post estimation
All categories	91.4	140.0	65.29	10,239	6685
Relevant categories	81.8	100.2	81.64	8328	6798

### Analysis

#### Emotion and Language Style

Language is the means through which thoughts are expressed, and it lies at the heart of human cognition and our ability to comprehend the world around us or, at the very least, to change and exchange that comprehension. The computer study of these comprehensions, feelings, emotions, evaluations, and attitudes regarding things, such as products, services, organizations, persons, issues, events, themes, and their characteristics, is known as sentiment analysis [31].

A tone analyzer service, such as the IBM Watson Tone Analyzer, detects anger, sadness, fear, and joy as emotions, and analytical, confidence, and tentative aspects as language styles in user inputs via text analysis [32,33]. The service can analyze tone at both the document and sentence levels in a document up to 128 KB and up to 1000 individual sentences per input. The Watson Tone Analyzer has an API that receives text as input and returns the tone at the sentence and document levels. In our case, the document level makes sense as we want to evaluate the tone of each post at once.

#### Topic Similarity and Difference

Social media text analysis employs a broad range of approaches or algorithms to process language, one of which is topic analysis, which is used to automatically discover a group of words (ie, a topic) from text. The literature investigates 2 types of topic analysis approaches. The first is topic modeling, which uses unsupervised models to find hidden topics in document collections, such as latent Dirichlet allocation [34] or Probabilistic Latent Semantic Visualization [35]. The second is topic classification, which uses supervised topic models based on document collection labels [36]. Recently, topic modeling has been used to compare the content of 2 document corpora. For example, Oelke et al [24] have developed a visualization approach to represent topics from 2 documents in a 2D space. Ren et al [25] have proposed methodologies to identify semantic commonality and distinction across a set of documents. ContraVis [23] involved a supervised joint approach for visually representing documents and associated topics in a 2D space. We selected ContraVis to compare the topics and perform content analysis of posts on Steemit and Reddit for 2 reasons. First, the ContraVis code was readily available and working from GitHub [23], and second, ContraVis represents the most recent work on topic comparison across corpora.

#### Clickbait or Nonclickbait

The term clickbait refers to using alluring headlines that employ writing formulas and linguistic methods to “bait” readers into clicking items [34]. Even though media scholars continually portray clickbait material in a negative light [37], the business built on it has been quickly developing and reaching an increasing number of individuals across the world [38]. News organizations have shifted to a digital front, in part, to stay afloat. They usually generate revenue via (1) advertisements on their websites or (2) a subscription-based model for articles that readers might be interested in. Writers have started using clickbait to attract more readers and boost the number of clicks on their material, therefore raising their agency’s revenue [39]. In the internet age, every media organization must compete with other media outlets for reader attention, and readers’ clicks are how they make money. Clickbait is also considered an indication of user engagement [40]. For example, Bhowmik et al [40] have demonstrated that clickbait posts related to health care can actually increase user engagement. Given that we also dealt with health care posts in this research, we can assume that if a post is identified as clickbait by our approach, the post will have more user engagement.

### Online Survey

Crowdsourcing is the practice of collecting opinions or information from those who engage in a “crowd.” Amazon Mechanical Turk (MTurk) is a well-known crowdsourcing platform that has emerged in the last decade [41]. We conducted an online survey using MTurk to assess information quality in Steemit and Reddit posts.

### Privacy and Ethical Considerations

All the study data (the secondary data set from Steemit as well as the data from the user survey) were anonymized. The study was conducted under protocols approved by the University of South Florida Institutional Review Board (STUDY003306: “Investing the drivers of currency in blockchain social platforms”) under HRP-502b(7) Social Behavioral Survey Consent. The approval covered the use of the publicly available anonymized secondary data set of Steemit posts as well as the survey of users to evaluate the quality of both Reddit and Steemit posts. No individual-specific data were gathered even in the survey; the only information gathered was about the subjects’ opinions of the content of social media posts shown to them in the survey. The consent form was provided in a downloadable format to participants at the beginning of the survey, and they were allowed to withdraw at any moment. The participants in this survey received a US $1 reward, and participation was fully anonymous.

## Results

### Emotion and Language Style

In our analysis, we randomly selected 2000 posts, 1000 for each platform (Reddit and Steemit). Posts on social media are not cleaned texts as they have misspellings, URLs, emojis, etc. We first cleaned the text using the Python NLTK library to remove stop words, URLs, and any non-English words from the text. Then, we applied stemming and lemmatization to generate standardized words. Each cleaned post was submitted to the Watson API, and then, the document-level tones were stored as a result.

Tables 4 and 5 show the Watson Tone Analyzer results for emotion type and language style, respectively, in Steemit and Reddit. From Table 4, we can observe that Steemit posts primarily represent “joy,” which is the result of Steemit posts primarily providing information, tips, and solutions related to health care topics. Steemit posts also appear to be phrased more positively and enthusiastically. On the other hand, a high number of Reddit posts have emotional tones of sadness, fear, and anger. This may be the result of social media users using the Reddit platform to share their own personal experiences in health care and to look for additional support.

From a language style perspective, as Table 5 shows, the numbers of confidential and analytical posts were higher on Steemit than on Reddit, while the number of tentative posts was higher on Reddit than on Steemit. These differences suggest that users on Steemit try to write factual content to motivate others to vote for them. In contrast, Reddit writers mostly provide their opinions and experiences to share with others. In Table 6, we provide some examples of Steemit and Reddit posts that also highlight the differences in these platforms from the emotional and language style perspectives. While we did not formally evaluate the Watson Tone Analyzer here (not the goal of this work), the labeling appears to be performing well on these corpora based on our analysis of a subset we extracted for manual review (experts performed the manual review without knowing Watson’s results). The above analysis of Steemit and Reddit posts for emotion and language style indicates that most of the posts on Steemit are informative, whereas people share their personal experiences on Reddit.

**Table 4:** Watson Tone Analyzer results for emotion aspects.

Emotion aspects	Steemit posts (N=1000), n	Reddit posts (N=1000), n
Joy	435	200
Sadness	276	422
Fear	105	125
Anger	3	22

**Table 5:** Watson Tone Analyzer results for language style aspects.

Language style aspects	Steemit posts (N=1000), n	Reddit posts (N=1000), n
Confident	77	30
Analytical	619	430
Tentative	416	627

**Table 6:** Emotion and language style samples.

Platform and post		Emotion type	Language style
**Reddit**			
	I want to share this message along with my greetings and wishes for everyone to you guys. I wish universe, god bless u with peace, love, happiness and wealth. Meditation has changed my life, rewired my brain, I’m happier, loved, fulfilled than ever. I hope every single being who receive this positive frequency, to have a beautiful and fulfilling life, full of love to his/ her existence and to all living beings that share that beautiful universe with us. Peace and love. Namaste.	Joy	Confident
	I tried meditation January of this year to lessen my anxiety. I have been constantly meditating since then. But my head is still noisy and I still get pretty anxious. Yesterday there was a lot going on with work and I fell into a deep hole. I was shaking, my chest was tight, my head was aching and rushing with thoughts. I was anxious the whole day. It made me ask myself, how come I am still like this? I was full of judgment. I felt like me meditating is just play pretend. Is meditation not working for me?	Sadness and fear	Analytical and tentative
	What can I take that is safeish that will turn my brain off for two days. I want to sleep and dream and not answer my demanding life. Yes, I ne ed a vacation but not at option at this moment. I need a break from thinking. I’m not suicidal in the slightest and I just need to shut down. Thank you.	Anger	Analytical and tentative
**Steemit**			
	Thalassemia is a disease of anemia. About 8 to 10 thousand children are born in our country every year due to death of this disease. After a child comes to life after life, it is not seen in children with thalassemia. Dhaka is a life of depression. The dream of a mother with her child, the love of emotions disappears in the moment. It is possible to avoid such a tragic event if you are a little aware. Thalassemia treatment is extremely expensive. It has to continue the treatment throughout life. The permanent treatment...^a^	Sadness and fear	Analytical and tentative
	Turmeric has a strongly anti-inflammatory, anti-bacterial, anti-fungal action and contains antioxidants. It perfectly speeds up the healing and the exchange of the epidermis. It is also known as a remedy for discoloration and excessive pigmentation. How to take advantage of these amazing benefits of turmeric? In the form of a mask, of course :). Ladies in India have been doing this for ages! Making a turmeric mask is very easy - take two tablespoons of turmeric, mix with a bit of honey and buttermilk into...^a^	Joy	Confident and analytical
	A small disclaimer before I begin to rant: this post is from my perspective as I have seen and experimented in my country -Dominican Republic, also I have no intent to speak for every dominican ever, I'm not every dominican and also the flavor of health services I have mostly experimented - private - is different for what the majority uses -public- even though I know enough about public health in my homeland to rant enough about it too. With this covered up let me begin: My father is a very sick and fragile man so that means I've spent a lot of time in hospitals during...^a^	Sadness and anger	Tentative

^a^The text continues.

### Topic Similarity and Difference

The use of ContraVis on Steemit and Reddit document collections allowed us to discover hidden topics while also learning about common and discriminative topics within these collections. We also identified labels, documents, topics, and word clouds (as also done in ContraVis), including the top 20 words in each topic.

**Figure 3:**
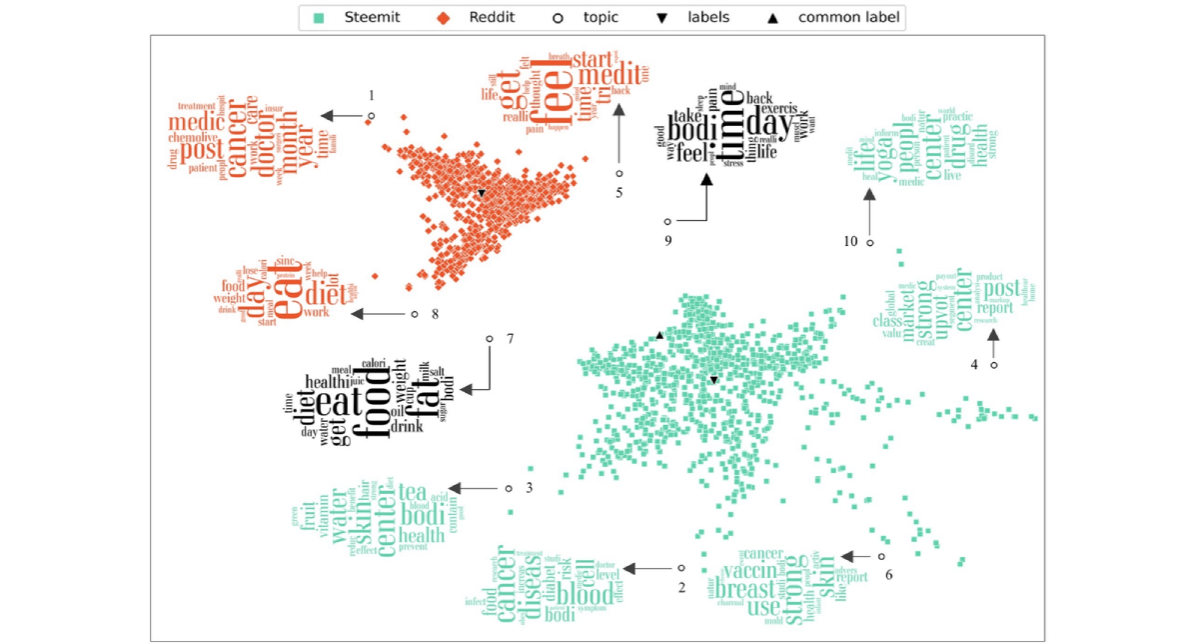
Contrastive visualization of Steemit and Reddit posts. The black clouds indicate the topics related to common topics across Steemit and Reddit, the turquoise clouds indicate topics in Steemit posts, and the orange clouds indicate topics in Reddit posts.

This procedure began with the compilation of 1000 posts for each social media platform, followed by removing stop words, stemming, and separating words in these 2000 documents. To create the word clouds, a vocabulary of unique terms and their indices were maintained, and the assembled documents were transformed from words to numbers as input in the ContraVis model. We set the number of topics in the ContraVis model to 10 since we gathered 10 health-related categories (health care, cancer, medication, etc) throughout the data collection process. The model generated coordinates for documents, topics, and labels. It also computed the probability of terms in each topic. As a result, we sorted the probabilities of words in descending order, used indices to match terms in the vocabulary file, and then visualized the word clouds. Furthermore, we have displayed the coordinates of documents, topics, and labels in Figure 3 to highlight topic similarities and differences in the Steemit and Reddit social media platforms.

As Figure 3 shows, more topics are placed around Steemit posts, indicating that Steemit posts are on more diverse topics than Reddit posts. This is also indicated by the scattered nature of Steemit posts compared with Reddit posts in the ContraVis visualization (Figure 3). Table 7 shows the number of posts associated with topic labels 1-10 shown in Figure 3.

According to Figure 3, each topic was associated with the word cloud. Focusing first on the common topics, we found that the 2 common topics represented food and nutrition, and exercise and mental wellness. It makes sense that these 2 topics were present in both since these could be informational (as is common on Reddit; driven by questions about how to eat healthily or be stress free, for example). In contrast, when we examined Steemit-specific topics, we found that some topics were more strongly informational in nature, without necessarily a personal angle. For example, one of the topics specific to Steemit loaded heavily on the words cancer, cell, blood, and disease. In contrast, the cancer-related topic on Reddit had greater weights for words like cancer, doctor, medicine, and chemotherapy, suggesting more discussion-oriented posts regarding the disease. In addition, we found many more topics associated with Steemit than Reddit, suggesting that the information diversity on Steemit may be higher. The results are also consistent with the finding that there are systematic differences in emotion and language style between the platforms, where the emotion/tone dimensions correlate with whether the content is a discussion or is social support oriented, or whether the content is mainly informational.

Thus, the content analysis of posts in this section also supported the conclusion from the previous section (Emotion and Language Style) that users post more informational content on Steemit, whereas Reddit posts are more personal in nature.

**Table 7:** Number of posts associated with each topic label.

Topic label	Number of posts	Platform
1	223	Reddit
2	154	Steemit
3	192	Steemit
4	37	Steemit
5	111	Reddit
6	262	Steemit
7	194	Common
8	515	Reddit
9	282	Common
10	30	Steemit

### Clickbait or Nonclickbait

Steemit is a cryptocurrency-based social media platform, where users gain Steem dollars for posting content that is valued by others. On the other hand, Reddit is primarily a traditional social media platform, where users mostly do not have any scope of personal economic gain for posts. Thus, we investigated whether Steemit users post more clickbait posts, which can increase user engagement, than Reddit users.

For detecting whether a post is clickbait, we used the following 2 approaches: (1) a manual approach, where clickbait content is identified by experts, and (2) a machine learning approach, where the manual approach is used for training a model and then the model is applied on a large number of posts.

For the machine learning model of clickbait detection, we referred to a previous report [27]. This model detects clickbait headlines using a neural network architecture based on Recurrent Neural Networks and relies on distributed word representations learned from a large unannotated corpus. Experimental results on a data set of news headlines showed that the model outperforms existing techniques for clickbait detection, with an accuracy of 98%. In this study, we implemented the model on 10,563 Steemit headlines and 876 Reddit headlines to calculate their clickbait percentages. At first, we manually labeled 600 headlines, of which 300 belonged to Steemit and the rest belonged to Reddit. Manual labeling was performed anonymously, preventing any possible bias for any of these platforms. The manual labeling results are presented in Table 8. In manual labeling, the Steemit headline clickbait percentage was about 2.5 times higher than the Reddit headline clickbait percentage.

In a previous report [27], the authors used a 50-50 data set to train the model; thus, we adopted the same approach but with oversampling to achieve a 50-50 balanced data set. We then trained the model based on our labeled headlines with various train-test proportions to detect clickbait headlines for the rest of our data set. For example, in the 75-25 train-test model, we used 75% of our oversampled labeled headlines for training and 25% for testing, making sure we had 50-50 clickbait and nonclickbait content in both the training and testing sets. As shown in Table 9, in the 75-25 train-test model, we achieved 96.45% accuracy in the testing set, with almost 21% (20.6%) clickbait percentage in total (2132/10263, 20.77% for Steemit and 101/576, 17.53% for Reddit). The clickbait percentage was higher for Steemit headlines than for Reddit headlines. We provide some samples of clickbait and nonclickbait posts from both Reddit and Steemit in Tables 10 and 11, respectively.

In conclusion, according to both manual labeling and clickbait detection model outcomes, Steemit headlines appeared to be more clickbait like than Reddit headlines. However, due to the unavailability of large training data, we could not determine the exact percentage of clickbait data in Reddit and Steemit. Intuitively, we could foresee that the reward-based incentive mechanism in Steemit may have motivated Steemit users to create more clickbait post headlines than Reddit users. However, our analysis does not allow us to draw any causal relationship between incentive mechanisms in social media and the existence of more clickbait post headlines.

**Table 8:** Manual labeling results.

Platform	Manual clickbait label, n	Manual nonclickbait label, n	Total, n	Clickbait percentage
Steemit	66	234	300	22
Reddit	26	274	300	8.67

**Table 9:** Clickbait detection model.

Model and platform			Model clickbait detection, n		Total headlines, n		Clickbait percentage		Model accuracy on the test set
**Train-test (75%-25%)**									96.45%
	Steemit	2132		10,263		20.77			
	Reddit	101		576		17.53			
**Train-test (80%-20%)**									~100%
	Steemit	1183		10,263		11.53			
	Reddit	58		576		10.07			
**Train-test (85%-15%)**									96.39%
	Steemit	1100		10,263		10.72			
	Reddit	60		576		10.42			

**Table 10:** Clickbait manual labeling samples.

Sample of clickbait headlines	Platform
How do you behave when you enter a foreign body in the eye?	Steemit
Early age hair fall cause	Steemit
Artificial Intelligence Can Predict How Much Longer You Have Left To Live	Steemit
Why do my eyes hurt during meditation	Reddit
Do you actually need 3 meals a day?	Reddit
Can a false positive urine drug test, in the end, reveal a false negative?	Reddit

**Table 11:** Nonclickbait manual labeling samples.

Sample of nonclickbait headlines	Platform
Flax-food or medicine?	Steemit
Exercise Best For Health	Steemit
Activated Charcoal for Skin Care	Steemit
Your diet/healthy eating peeps	Reddit
Liver failure due to cancer	Reddit
Nerve under my knee hurting?	Reddit

### Online Survey

We conducted an online survey using MTurk to assess information quality in Steemit and Reddit posts. We designed the study so that participants first read the post via a link that brought them to see the post on a third-party website without Steemit or Reddit logos, preventing possible biases in answering questions, and then answered 5 questions (mix of multiple choice and text entry types). This procedure was repeated 5 times for each participant. This means that by the end of the survey, each participant received 5 different posts, with the same 5 questions for each. Moreover, to score posts based on multiple responses, we assigned each post thrice to different participants. In this study, we recruited 276 MTurk employees and assigned 5 posts out of 460 random Steemit and Reddit posts (230 posts from Steemit and 230 posts from Reddit) to each and then asked the following questions after each post:

1. Compared to typical posts you see on social media, how good is this post in terms of content quality?
2. If you see this post in your feeds, how likely would you be to like or comment on this post?
3. If reading this post requires a subscription, would you pay money to subscribe?
4. Please copy and paste the most important sentence in the post.
5. Why do you think this sentence is the most important one?

The first 3 questions in the survey indicate the content quality. These 3 questions were multiple choice. The response options were “Good,” “Average,” and “Poor” for the first question; “Extremely likely,” “Neutral,” and “Not likely at all” for the second question; and “Yes” and “No” for the third question. The rest of the questions involved text entry. The purpose of the last 2 questions was to make sure participants read the assigned posts carefully. In the process of analyzing the results, we provided weights to each option (3 to “Good,” 2 to “Average,” and 1 to “Poor” for the first question; and 3 to “Extremely likely,” 2 to “Neutral,” and 1 to “Not likely at all” for the second question) based on the importance. For the final score, we obtained the maximum score of each post, and in case of a tie, we chose the worst option (eg, when a post equally scored “Poor” and “Average,” we chose “Poor”). Table 12 shows the number of posts distributed within each option based on the survey results. Most of the posts were classified into “Average” or “Good” categories, illustrating a high probability of having informative/interesting content, and according to Table 12, most of the Steemit posts were placed in these categories.

Table 13 shows the distribution of the likelihood to comment on or like posts based on survey results. The frequency of Steemit posts in the “Extremely likely” option illustrated that Steemit posts have the potential to be more informative/interesting compared with Reddit posts, so people are more willing to like the post or leave a comment.

Table 14 shows the results of the third question, which asked whether people are willing to subscribe to such posts. Based on the survey results, the probability of subscription was higher for Steemit posts than for Reddit posts.

We next assessed statistically whether the difference in the number of people who rated “Poor” (for example) in Steemit versus Reddit was significant. We did this for all classifications by MTurk users and tested whether the number of people who picked a certain value (eg, poor, extremely likely to comment, etc) was statistically different across a sample of Reddit and Steemit posts. For each classification, we tested the null hypothesis (*H_0_*: *μ_Steemit_*=*μ_Reddit_*; ie, equality of the means of the 2 groups) against the alternative hypothesis (*H_1_*: *μ_Steemit_*≠*μ_Reddit_*). The results are presented in Table 15. The tests’ *P* values for the “Poor,” “Average,” and “Good” options were far less than the significance level (*α*=.05) (Table 15). This implied that the content quality of posts on the Steemit platform was statistically different from that of posts on the Reddit platform. Moreover, Cohen *d* showed that the difference between the 2 means was large for the content quality question. For the question regarding the likelihood to comment on or like posts, the tests’ *P* values for the “Not likely at all” and “Extremely likely” options were less than the significance level (*α*=.05), demonstrating a statistically significant difference in the likelihood to comment on or like posts between the Steemit and Reddit platforms. On the other hand, the “Neutral” option’s *P* value was greater than the significance level (*α*=.05), showing not enough evidence to reject the null hypothesis. Finally, the *P* values for the “Yes” and “No” options implied statistically significant differences in the likelihood of subscribing to posts between the Steemit and Reddit platforms.

To summarize, regarding the content quality question, the number of people who picked the “Poor” or “Average” option was significantly higher for Reddit posts (mean_poor_ 0.996, SD_poor_ 1.055; mean_average_ 1.400, SD_average_ 0.987) than for Steemit posts (mean_poor_ 0.400, SD_poor_ 0.721; mean_average_ 1.165, SD_average_ 0.948; *P*<.001 and *P*=.01, respectively), whereas the number of people who picked the “Good” option was significantly lower for Reddit posts (mean 0.604, SD 0.833) than for Steemit posts (mean 1.435, SD 1.087; *P*<.001). Regarding the question involving the likelihood to comment on or like posts, the number of people who picked the “Not likely at all” option was significantly higher for Reddit posts (mean 1.435, SD 1.153) than for Steemit posts (mean 0.913, SD 1.003; *P*<.001), whereas the number of people who picked the “Extremely likely” option was significantly lower for Reddit posts (mean 0.600, SD 0.489) than for Steemit posts (mean 1.135, SD 1.095; *P*<.001). However, the number of people who picked the “Neutral” option did not differ between Reddit posts (mean 0.965, SD 0.984) and Steemit posts (mean 0.952, SD 0.873). Finally, regarding the subscription question, the number of people who intended to subscribe was significantly lower for Reddit posts (mean 0.722, SD 0.935) than for Steemit posts (mean 1.374, SD 1.152; *P*<.001), whereas the number of people who intended not to subscribe was significantly higher for Reddit posts (mean 2.270, SD 0.942) than for Steemit posts (mean 1.626, SD 1.152; *P*<.001).

**Table 12:** Number of posts classified in each option based on the first question results.

Content quality question	Steemit posts (N=230), n	Reddit posts (N=230), n
Poor	17	67
Average	81	106
Good	132	57

**Table 13:** Number of posts classified in each option based on the second question results.

Likelihood to comment on or like posts question	Steemit posts (N=230), n	Reddit posts (N=230), n
Not likely at all	55	95
Neutral	56	65
Extremely likely	119	70

**Table 14:** Subscription probability for Steemit vs Reddit posts.

Subscription probability	Steemit (N=230), n (%)	Reddit (N=230), n (%)
No	119 (51.7)	183 (79.6)
Yes	111 (48.3)	47 (20.4)

**Table 15:** Independent samples *t* test for the comparison of the null hypothesis to the alternative hypothesis.

Question and options		*t* test (*df*)	Alternative	*P* value	95% CI	Cohen *d*	Power
**Content quality question**							
	Poor	−7.067 (458)	2-sided	<.001	−0.76 to −0.43	0.659	1
	Average	−2.602 (458)	2-sided	.01	−0.41 to −0.06	0.243	0.738
	Good	9.196 (458)	2-sided	<.001	0.65 to 1.01	0.858	1
**Likelihood to comment on or like posts question**							
	Not likely at all	−5.178 (458)	2-sided	<.001	−0.72 to −0.32	0.483	0.999
	Neutral	−0.150 (458)	2-sided	.88	−0.18 to 0.16	0.014	0.052
	Extremely likely	5.851 (458)	2-sided	<.001	0.36 to 0.71	0.546	1
**Subscription question**							
	No	−6.558 (458)	2-sided	<.001	−0.84 to −0.45	0.612	1
	Yes	6.667 (458)	2-sided	<.001	0.46 to 0.84	0.622	1

### Summary

Integrating the findings across all the results presented above, we found that health-related content on incentive-based social media platforms seemed more informational rather than discussion oriented or personal. Moreover, incentive-based platforms appear to encourage their content providers to post higher-quality content, but with more attention-grabbing headlines. Table 16 summarizes these findings.

**Table 16:** Summary of the findings.

Dimension	Main result	Conclusion
Topic modeling	Only 20% of all topics were common. Steemit topics were more informational.	Steemit users post more informational content, whereas Reddit posts are more personal in nature.
Emotion and language style	Emotion: Steemit - Joyful content Reddit - Sad, fearful, and angry content Language style: Steemit - Confident and analytical content Reddit - Tentative content	Because posts are more informative on Steemit, the language styles and emotions are more positive.
Clickbait	Steemit headlines were more likely to be clickbait than Reddit headlines.	The reward-based incentive mechanism may have motivated users to create more clickbait headlines.
Content quality	Steemit posts had better quality than Reddit posts. Users were more likely to like, comment on, or subscribe to Steemit posts than Reddit posts.	Posts from the incentive-driven platform were likely to be seen as having higher quality.

## Discussion

### Principal Findings

The main objective of this study was to understand differences in health-related social media content across platforms with and without monetary incentives. Our methodology, as noted above, combined machine learning techniques (topic modeling and sentiment analyses) with human survey results and examined differences across emotion and language style, topic similarity and difference, whether the post was clickbait, and content quality as assessed subjectively by users.

The IBM Watson Tone Analyzer API highlighted important differences in both language style and emotion across the Steemit and Reddit social media platforms. In terms of language style, the Watson Tone Analyzer service identified posts as confident, analytical, or tentative (or a combination if relevant). Using a sample of 2000 posts from Steemit and Reddit, we found that more than double the number of Steemit posts had a confident language style compared with Reddit posts (specifically, 77 posts from Steemit and 30 from Reddit were scored as “confident”). Steemit scored higher again for analytical content, and 50% more Steemit posts were identified as having analytical content (specifically, 619 posts from Steemit and 430 from Reddit were scored as “analytical”). On the other hand, 33% less Steemit posts had a tentative language style (specifically, 416 posts from Steemit and 627 from Reddit were scored as “tentative”). In terms of emotion, the Watson Tone Analyzer service labeled posts as joy, sadness, fear, or anger (or a combination if relevant). When provided with the same sample of 2000 posts from Steemit and Reddit, we found that more than double the number of Steemit posts were scored as having a joyful emotion compared with Reddit posts (specifically, 435 posts from Steemit and 200 from Reddit were scored as “joy”). For the other 3 dimensions, Reddit posts seemed more likely to have such content. Specifically, for sadness, there were 53% more Reddit posts than Steemit posts (422 from Reddit and 276 from Steemit). Moreover, for fear, there were 19% more Reddit posts than Steemit posts (125 from Reddit and 105 from Steemit). Furthermore, for anger, there were 22 posts from Reddit compared to only 3 from Steemit.

Our analysis of similar and different topics using the contrastive topic modeling platform ContraVis showed important differences as well. The use of ContraVis on 1000 randomly selected posts each from the 2 different platforms showed that only 20% of all topics were common (2 common topics out of 10). In particular, topics like “food and nutrition” and “exercise and mental health” were common on both platforms. Steemit had more unique topics than Reddit (5 vs 3), and those were more informational in nature rather than discussion oriented, as was the case for Reddit posts.

All the findings together suggest that posts from the incentive-driven platform were more likely to be informational and optimistic in nature, while posts from the traditional social media platform were likely about individual experiences and the discussions such experiences generate on social media.

When we analyzed these data from a “clickbait” perspective, we found that overall more Steemit posts were likely to be categorized as clickbait compared with Reddit posts, suggesting that incentive-driven platforms may encourage authors to compose content that will seem attractive to users. According to the clickbait findings, manual labeling marked more Steemit headlines as clickbait than Reddit headlines (66 vs 26), and a machine learning model that was trained to detect clickbait also labeled a higher percentage of Steemit headlines as clickbait than Reddit headlines.

Finally, in the user survey, MTurk users said that at least 57% of Steemit posts had better quality than Reddit posts, and MTurk users were at least 52% more likely to like and comment on Steemit posts rather than Reddit posts. These findings suggest that posts from the incentive-driven platform were likely to be seen as being of higher quality, which is an important observation as well.

### Implications

As incentive-based social media ideas gradually enter the mainstream, it becomes increasingly critical to study how incentive systems built into these platforms influence the type of material created on social media platforms. Could these systems aid in the generation of higher-quality data? As we have seen globally, social media plays a massive part in people’s lives, but it continues to pose numerous information quality issues, not the least of which is the growing worry about fake news in the context of health (eg, vaccination-related content [42]). As an important step in that direction, in this study, we systematically compared content from 2 social media platforms (the nonincentive-based social media platform Reddit and the incentive-based social media platform Steemit) in terms of topic modeling, emotion, language style, and clickbait. Given the recent relevance of this issue in the context of health disinformation concerns on social media, we focused on health-related posts on these platforms.

While the incentive-based Steemit platform is new, there is growing interest in understanding this better. There has been some early work, for instance, that studied the Steemit platform from the perspectives of decentralization, reward mechanisms, and user behavior. In previous work [13], the foundations of decentralized content curation were studied from a computational perspective. A model was developed under different scenarios to understand how the Steemit system curates arbitrary lists of posts. The results showed that Steemit’s voting power mechanism and the possibility of self-voting might induce selfish behavior across users. Our research was a continuation of that phenomenon and focused on the users’ content. Thelwall [12] and Guidi et al [14] studied the sentiment and topic effect on post rewards for a user’s first post on Steemit and discussed how sentiment affects the success of posts and the post topic influences popularity. We extended that discussion considering all Steemit posts for health topics (neglecting their published date). Additionally, we compared the sentiment, topic, clickbait, and quality of posts between an incentive-based platform (Steemit) and a nonincentive-based platform (Reddit). Guidi et al [15] studied the impact of the witness mechanism on the Steemit platform. While insights about content on Steemit related to how sentiment, topics, and social capital play roles have been studied in past literature [12,15], formal comparisons with other platforms like Reddit were limited (it was done mainly conceptually and not experimentally). In this research, we analyzed posts from these 2 platforms more holistically across several dimensions. Our research provides insights into the implications of incentive mechanisms for social media content.

In particular, we did find evidence that the incentive-based mechanism may be leading social media users to provide more informational content, which may also be more diverse and with carefully constructed titles to help generate engagement. In some ways, this partly resembles how the mainstream news media have evolved as the shift to digital platforms forced many of them to present content in a manner that engages users. Unlike some mainstream media, the articles themselves appeared to be more informational, perhaps guided by user expectations that such content may be more likely to generate votes from the community, leading to the potential of greater cryptocurrency rewards. We did not assess causality explicitly in this study, and therefore, we suggest this as a possible explanation but not an established empirical observation yet. Quite interestingly, we did find the tone of messages to be quite positive on Steemit, suggesting that users are not necessarily resorting to fear or other negative emotions to garner engagement.

In comparison, we did find that conventional social media (Reddit) does contain more personal stories and discussions, making this perhaps a better place for users who come for input or support from the community. Reddit has recently introduced its own cryptocurrency (Moon), and our results here should suggest some caution since greater adoption of reward-based schemes may take away the valuable aspect of support communities existing today on platforms such as Reddit. We are starting to see unsurprisingly that incentives do affect user behavior, and greater adoption of this by social media platforms may turn the average social media user into a “citizen journalist” battling for eyeballs and engagement.

While our comparison was more exploratory in nature, rather than guided by specific directional hypotheses, we believe that the systematic comparison performed here is one of the first such studies and therefore represents an important contribution. The findings, as noted above, have significant implications for the intended design of next-generation social media. Platforms can take advantage of reward mechanisms to gain more engagement and high-quality informational content on diverse topics. We do see some of the values that can come from incentive mechanisms, but also see evidence that a greater focus on this may negatively impact the community and the social support–related functions that these media provide.

### Limitations and Future Work

This study has important limitations. As mentioned before, the platforms may be different in many dimensions, and in this study, we only focused on some important dimensions. However, there are other important aspects, notably misinformation and fake news, that need to be examined across incentive and nonincentive-based platforms in future work. Moreover, there is some information on these platforms that we do not have access to, specifically the network structure of the relationships among users, and consequently, we did not study the differences because of them. Further, the platforms we compared were different in terms of how long users participated. Although Steemit is a new social media platform compared with Reddit, it has been a very active and important platform, as it has 1,643,143 registered accounts, and within the first 45 months of its launch (March 24, 2016), 17,805,355 new posts were published on this platform. Finally, this is an exploratory study and does not provide specific causal interpretations. We hope future work can systematically address some of these limitations to build on this potentially important research direction for researchers.

There are many opportunities for future work, and we highlight a few here. First, extending our exploratory analyses to establish more formal causal links would be necessary for major policy decisions. Second, expanding both the categories and the types of social sites compared (eg, Facebook and Twitter) will make the findings more nuanced. Third, a longitudinal analysis of these platforms to study threads of discussions can present a more thorough comparison as well and is something that can be studied through recent deep learning models. Fourth, examining the other components of incentive-based social media (other than the incentive mechanism) would also be interesting. For example, would the permanency associated with blockchain-based systems affect how users participate in such media? Fifth, examining misinformation and fake news separately in the different platforms to study how they differ could be an important contribution as well.

### Conclusion

This study is the first to compare an incentive mechanism–based platform against a traditional platform systematically. We compared health-related posts on 2 social media platforms using machine learning and statistical analysis tools, and found differences in examined dimensions (ie, emotions and language styles, topic similarity and difference, clickbait and nonclickbait headlines, and content quality). Our findings demonstrate that the incentive mechanism was associated with more informational posts on diverse topics, whereas posts from the traditional social media platform were more likely about individual experiences in a discussion format. Our user survey results also showed that posts from the incentive-based platform were of higher quality. It also suggested that users on the incentive-based platform, perhaps because of the rewards, make their headlines more clickbait like to an extent to encourage more engagement.

Social media has radically altered how the world distributes and receives health care information. One example may be the COVID-19 pandemic, which emphasized the value of social media as an influential information (could be misinformation or disinformation) source and demonstrated how it affects care on a variety of levels [43]. As another example, since the pandemic continues impacting people on personal levels, people tend to care more about health news on social media [44]. However, distinguishing high-quality health information from low-quality health information is a major problem on social media platforms (eg, disinformation and misinformation) [10,45,46], and remains an issue that has not been sufficiently addressed in health care communities. According to a study [47], social media users (638/1003, 63.6%) were less likely to confirm what they read online with a doctor, which highlights the importance of information quality on such platforms. Sadly, the sheer volume of material being regularly posted makes any kind of real-time fact-checking or verification impossible [43]. Determining who is accountable or liable, as well as how ethics, privacy, confidentiality, and information quality should be controlled, will continue to be crucial issues that need to be resolved [48]. Our research adds an important angle to previous work [43-46] in health care social media by exploring a possible way to address health information quality on social media. We explored this possibility by comparing health care posts across 2 social media platforms where the main difference was the existence of an incentive-based system.

Our theoretical contribution shows that the incentive structure in social media can affect specific characteristics of the content of health care social media posts. The practical implication of our work is that the design of future social media platforms targeted toward health care should explicitly consider developing incentives for users as a mechanism to help content quality. A better internet environment for social networking, collaboration, participation, apomediation, and openness [6] is a key concept of Web 2.0, and Medicine 2.0 was developed to respond to the advent of Web 2.0. To this end, social media are essential platforms for these concepts, and an incentive-based platform can contribute to these. Incentive-based platforms can be a way to distinguish high-quality health information [9] from low-quality information (eg, online misinformation) [10]. Moreover, they can be effective tools for those who are looking for health information [6-8] and can play a role in the next generation of Web 3.0 platforms for health information. However, we caution that these are early days for incentive-based social media platforms, and more work is needed to understand not just the direct effects but also some second-order effects. For example, it is possible that incentives for content and participation could skew the content toward certain categories more than others. For example, at the inception of Facebook, not many people could imagine that a social network designed for Harvard students could change how individuals interact with one another on a global scale. In health care, it is also possible that the content generated becomes focused on areas with more need (and therefore more users), potentially hurting niche topics, for example. Thus, from a research perspective, there is great potential for developing new insights that can guide the proactive design of next-generation social media platforms and online health communities.

## Abbreviations

API: application programming interface
MTurk: Amazon Mechanical Turk

## Data Availability

The data sets generated during or analyzed during this study and the source codes are available in the GitHub repository [49].
